# Anti-VEGF treatment is the key strategy for neovascular glaucoma management in the short term

**DOI:** 10.1186/s12886-016-0327-9

**Published:** 2016-08-30

**Authors:** Yaoyao Sun, Yong Liang, Peng Zhou, Huijuan Wu, Xianru Hou, Zeqin Ren, Xiaoxin Li, Mingwei Zhao

**Affiliations:** 1 Department of Ophthalmology, Peking University People’s Hospital, 11 Xizhimen South Street, Xi Cheng District, Beijing 100044 China; 2 Key Laboratory of Vision Loss and Restoration, Ministry of Education, Beijing, China; 3 Beijing Key Laboratory of Diagnosis and Therapy of Retinal and Choroid diseases, Beijing, China

**Keywords:** Neovascular glaucoma, Anti-VEGF therapy, Pan-retinal photocoagulation, Anti-glaucoma surgery

## Abstract

**Background:**

To present a comprehensive approach for the management of patients with neovascular glaucoma (NVG) aiming to preserve visual function and complement pan-retinal photocoagulation (PRP) by anti-vascular endothelial growth factor (anti-VEGF) treatment and anti-glaucoma surgery.

**Methods:**

This study includes a prospective, interventional case series. A process flow chart for NVG management was designed. Totally 50 patients (51 eyes) with NVG were included. Of these, 43 patients (44 eyes) completed the treatment process. Patients were divided into central retinal vein occlusion (CRVO) and proliferative diabetic retinopathy (PDR) groups according to their original diagnosis. Intraocular pressure (IOP), visual function, and the status of iris and angle neovascularization were recorded before and after treatment.

**Results:**

Patients were followed up for 6–30 months (mean 12.2 months). The IOP of all 44 patients was effectively controlled and was significantly less after treatment (16.68 ± 4.69 mmHg) than before treatment (42.59 ± 9.44 mmHg, *P* < 0.05). Thirty-nine eyes displayed controlled IOP (≤21 mmHg) after treatment. Visual acuity improved, to some extent, in 32 eyes (72.9 %), and 12 eyes (27.3 %) had a visual acuity better than 0.1. There was no significant difference in IOP between the PDR and CRVO groups at the end of follow-up (*P* = 0.8657), but the visual acuity in the PDR group was much better than that in the CRVO group (*P* = 0.0079).

**Conclusions:**

A comprehensive therapy for NVG can effectively control IOP and preserve visual function in patients by anti-VEGF injection and anti-glaucoma surgery.

## Background

Neovascular glaucoma (NVG), a common type of refractory glaucoma, is closely related to retinal ischemia, which is usually secondary to proliferative diabetic retinopathy (PDR) and central retinal vein occlusion (CRVO). Vascular endothelial growth factor (VEGF) is usually released after retinal ischemia, and it can spread through the aqueous humor to the anterior segment of the eye. This results in the neovascularization of the iris, angle, and connective tissue membrane and is followed by synechia of the peripheral iris and trabecular meshwork, which can ultimately cause increased intraocular pressure (IOP) or even loss of vision [1–3].

Treatment for NVG is far from satisfactory due to the complexity of the primary disease as well as the specificity of NVG itself. Its onset is abrupt and induces IOP to increase by a large margin, which will cause a loss of visual function if not controlled as soon as possible. Commonly used anti-glaucoma drugs do not effectively decrease IOP in NVG, and traditional anti-glaucoma surgery is also infeasible because there is a large abundance of neovessels on the surface of the iris and angle, and damaging these vessels can lead to intraoperative bleeding during surgery. In addition, damage to the blood-aqueous barrier and the leakage of plasma proteins are likely to stimulate the fibrovascular membrane to grow into the filtration egress, which will ultimate lead to the failure of filtration surgery. Moreover, the effect of glaucoma valve implantation is also not satisfactory. Although cyclocryotherapy and cyclophotocoagulation can effectively decrease IOP, difficulties exist in their precise quantitation and ocular atrophy and loss of vision eventually occur in many of these patients [4–7]. Another currently popular treatment for NVG is the injection of intravitreal anti-VEGF antibodies. This is expected to directly decrease the level of intraocular VEGF and to cause the regression of neovascularization in the retina, angle, and iris. In addition, intravitreal bevacizumab (IVB) is currently widely used to prevent the formation of irreversible synechia in early rubeosis and to control IOP in patients with iris neovascularization (INV) alone and in those with early stage NVG without angle closure [5, 6, 8–10]. However, there is still no consensus regarding the optimal management strategy for NVG.

The core of the problem lies in determining what the goal of NVG treatment should be: to decrease IOP or to preserve visual function. Is it possible to abandon the use of cyclocryotherapy or cyclophotocoagulation, which are destructive treatments, in an affected eye that still has visual functions [4, 11–13] ? Which treatment is key to treating NVG: anti-VEGF medication, anti-glaucoma surgery or complete pan-retinal photocoagulation (PRP)? Many questions remain to be answered.

Therefore, we conducted this prospective, single center, interventional case series study to establish a comprehensive treatment strategy for NVG, with a core aim of preserving visual function and a main goal of completing PRP. Anti-VEGF injection and anti-glaucoma surgery were used as the main methods to preserve visual function and to control IOP.

## Methods

### Patients

An informed consent process was established following the guidelines of the Helsinki Declaration, and written informed consent forms were signed by all subjects to participate in this study. The study was approved by the medical ethics committee of Peking University People’s Hospital.

Fifty NVG patients (51 eyes) were recruited in this consecutive case series study between Jan. 1, 2010 and Dec. 31, 2012 in Department of Ophthalmology, Peking University People’s Hospital. Forty-three patients (44 eyes) were followed up for at least 6 months after the treatment process. Participants included 29 males (30 eyes) and 14 females (14 eyes) and ranged from 18 to 77 years old (mean age of 49.95 years old). Nineteen patients (19 eyes) had CRVO, and 24 patients (25 eyes) had PDR. Patients were divided into CRVO and PDR groups according to their original diagnosis, but not for the purpose of this study. All patients were followed up for at least 6 months after the last surgery, and patients without surgical treatment but with a stable IOP were also followed up for at least 6 months. Patients with residual glaucoma but with IOP controlled by an ocular hypotensive medication were followed up for at least 3 months. The inclusion criteria for the patients were 1) NVG was caused by retinal vascular disease, 2) an IOP ≥ 24 mmHg and 3) iris and angle neovascularization, angle open or closed. NVG patients who previously received cyclocryotherapy or cyclophotocoagulation or in whom NVG was caused by non-single retinal ischemia, such as ocular ischemic syndrome, were excluded from this study. A visual acuity measurement was obtained for each patient according to the International Standard Visual Acuity Chart, and IOP was measured in each patient using a Goldmann applanation tonometer. A 360°angle examination was carried out under a gonioscopy at the initial examination. NV of the angle can range from fully open, partly open or complete synechia closure. Sometimes, however, the details of the angle were hard to tell because of the corneal edema caused by the high IOP.

### Decrease IOP

Paracentesis of the anterior chamber is the premier treatment for quickly decreasing IOP. A 26-gauge needle was applied to puncture the anterior chamber at the inferior temporal cornea limbus parallel to the iris. This avoided hemorrhage due to quickly decreased IOP. The aqueous was slowly released until the neovascularization dilated and oozed. The anti-glaucoma drugs used included: carbonic anhydrase inhibitor (orally or locally), β-blocker, α-agonist and prostaglandin (locally). For patients at the early stage of NVG, 500 ml of 20 % mannitol was also applied.

### Intraocular anti-VEGF injection

An intraocular bevacizumab was carried out immediately after diagnosis to decrease the VEGF concentration in the vitreous. Bevacizumab (0.05 ml/1.25 mg, Roche, Switzerland) was administered in this study as an anti-VEGF medication. Intravitreous injections were performed according to previously reported methods [1, 9]. If a repeated treatment was necessary, it was administered 4 weeks after the previous treatment [14].

### Anti-glaucoma surgery with or without phacoemulsification or vitrectomy

The initial method used for anti-glaucoma surgeries in the study patients was trabeculectomy, as previous described and mitomycin C were used in trabeculectomy [3, 6]. When combined with a vitrectomy, the scleral flap was made beforehand, and a 23G incision or a 20G manual small incision vitrectomy technique was applied during intraocular surgery and was followed by an anti-glaucoma surgical procedure [15, 16]. When combined with cataract surgery, routine phacoemulsification and intraocular lens implantation were performed through the same incision before trabeculectomy and iridectomy.

### Pan-retinal photocoagulation

PRP was performed under a slit lamp using a U.S. LUMENIS multi-wavelength fundus laser machine. For those patients who reveived vitrectomy surgeries, an introcular 810 infrared laser was used for photocoagulation during the surgery, if necessary.. A level II to III reaction for retinal photocoagulation was appropriate for laser output power intensity, the spot size was 300 microns, the exposure time was 0.3 s, and the photocoagulation scope was 0.5–1 papilla diameter (PD) on the nasal side in the optic disc and 2 PD on the temporal side in the macula and the left retina outside of both the up and down vascular arcades. The choice of the laser wavelength was based on the degree of the refractive media opacity. A red laser was preferred for photocoagulation when refractive media opacity or flame-shaped hemorrhage due to CRVO was present. If PRP could not be conducted due to refractive media opacity, cataract surgery or vitrectomy combined with intraocular photocoagulation (with or without glaucoma surgery) were performed under the conditions used for the previous anti-VEGF treatment. If photocoagulation could not be completed because of hemorrhaging in the retinal surface, anti-VEGF therapy was repeated every month until the blood was absorbed to complete photocoagulation. If there was no significant refractive media opacity or bleeding on the retinal surface, photocoagulation was divided into two treatment periods of 500–700 points each with an interval of 3–7 days. Whether supplemental photocoagulation was necessary depended on the results of analysis by fundus fluorescein angiography (FFA) at 4 weeks after the first PRP. The setting for total photocoagulation was 1500–2500 spots.

### Statistical analyses

Data analysis was performed using SPSS software (version 10.0). Statistical comparisons between the CRVO group and the PDR group IOP measurements before and after treatment were calculated using Student’s *t*-test, while visual acuity comparisons between the two groups before and after treatment were calculated using chi-squared tests.

## Results

**Fig. 1:**
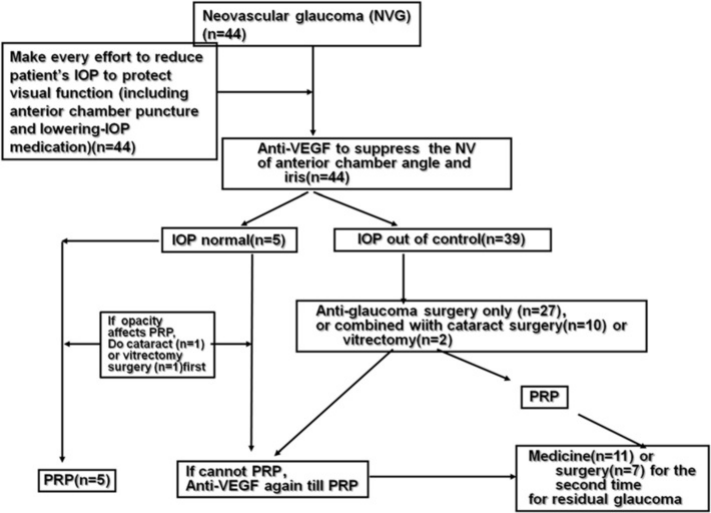
A chart of the neovascular glaucoma treatment process

In this prospective, interventional case series, the completion of the treatment process is demonstrated in Fig. 1.

After all efforts to decrease IOP were performed, 39 (88.6 %) of the eyes with uncontrolled IOP received trabeculectomy with or without phacoemulsification or vitrectomy. Although 7 (17.9 %) of the eyes had received a glaucoma valve implantation for a second time and 11 (28.2 %) eyes required anti-glaucoma drug treatment due to residual glaucoma, conditions were created so that all 44 (100.00 %) of the patients completed PRP using the total laser amount, which was 1500–2800 points. FFA showed no neovascular leakage. Finally, all of the patients finished the treatment process.

After the completion of the treatment process, the IOP of 39 (88.6 %) of the eyes was effectively controlled (≤21 mmHg), and the patients were followed up for at least 6 months. The IOP of the other 5 (11.4 %) eyes was between 21 and 28 mmHg. This difference was significant and was calculated between the mean preoperative IOP (42.59 ± 9.44 mmHg) and postoperative IOP (16.68 ± 4.69 mmHg) (*t* = 17.07, *P* < 0.05).

**Fig. 2:**
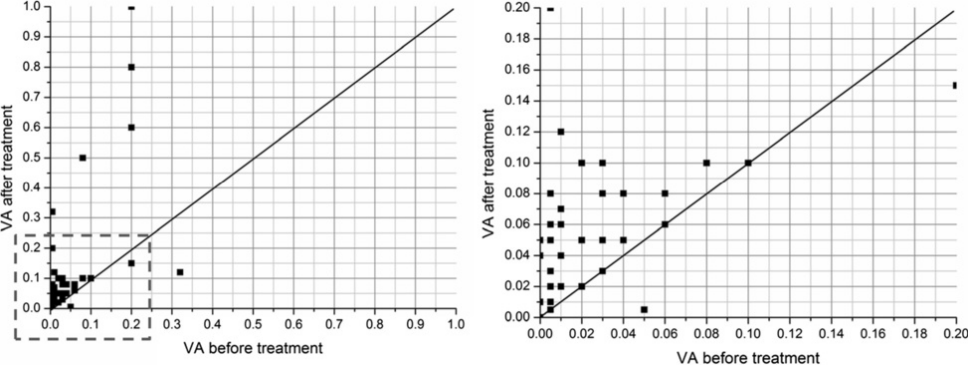
Visual acuity before and after treatment. A total of 32 (72.9 %) eyes showed improved vision, to various degrees. Of these, 12 (27.3 %) had a visual acuity ≥0.1, 9 (20.5 %) remained unchanged, and 3 (6.8 %) became worse. Of the NLP eyes at the time of diagnosis, 3 of 5 had re-acquired their visual acuity at the end of treatment and had visual acuity values of 0.05, 0.04, and 0.01

Because the core aim of this study was to preserve visual function, after the treatment process, 32 (72.9 %) of the eyes showed improved visual acuity, to various degrees, and 12 (27.3 %) of these had a visual acuity ≥ 0.1, 9 (20.5 %) remained unchanged, and 3 (6.8 %) became worse (Fig. 2). Three out of five eyes with no light perception (NLP) at the time of diagnosis showed restored visual acuity at the end of treatment, with visual acuity values of 0.05, 0.04, and 0.01.

Although residual neovessels were still observed on the surface of the iris in some cases, neovascularization of the iris and angle had clearly regressed in all of the patients after completion of the treatment process. Angle examination was not conducted in most cases due to the presence of increased IOP, corneal edema, or severe symptoms of pain prior to treatment in this group. A postoperative angle check was performed on 22 eyes, and these showed angle openings greater than half of the circumference in 9 cases. Of these, 3 eyes required anti-glaucoma medication after the last follow-up. The angle of the remaining 13 eyes was partially or totally shut off.

### Surgery complications and management

Bleeding occurred in all patients who received a trabeculectomy when an iridoctomy was performed. Massive bleeding did not occur as a result of preoperative anti-VEGF treatment. Seven eyes (17.9 % of the 39 eyes that received a trabeculectomy) suffered from a mild shallow anterior chamber and choroidal detachment after the trabeculectomy, a condition that was recovered 7–10 days postoperatively through topical steroid and cycloplegic treatment. Seven eyes had mild anterior chamber bleeding that regressed within 1 week after the operation. One eye that received a vitrectomy combined with a trabeculectomy suffered from vitreous hemorrhage, and this was absorbed within 1 month. In patients who received a glaucoma valve implantation, one case had hyphema, which was absorbed within 2 weeks.

Eyeball massage was performed in five cases on the first day after the trabeculectomy due to elevated IOP because of a small amount of bleeding at the filtration pathway. In seven other cases with a shallow anterior chamber and mild choroidal detachment, massage was started 7–10 days postoperatively when the anterior chamber formed and the choroidal detachment recovered, and these patients were taught how to self-massage after discharge. Filtration blebs became unobvious in 19 (48.7 %) eyes 3 months after surgery, and 7 (17.9 %) eyes with an increased IOP that could not be controlled received a valve implantation for a second time.

### Comparisons between the CRVO group and the PDR group

The baseline IOP was 45.58 ± 8.15 mmHg in the CRVO group and 41.04 ± 8.95 mmHg in the PDR group, which was not a statistically significant difference (t = 1.73, *P* = 0.09). The IOP after the last follow-up was 17.53 ± 5.21 mmHg in the CRVO group and 16.04 ± 4.25 mmHg in the PDR group, with no statistically significant difference (t = 1.04, *P* = 0.30). Baseline visual acuity in the CRVO group was NLP to counting fingers (CF) in 10 (52.6 %) eyes, 0.01–0.09 in 8 (42.1 %) eyes, and better than 0.1 in 1 (5.30 %) eye. Baseline visual acuity in the PDR group was NLP to CF in 8 (32.0 %) eyes, 0.01–0.09 in 12 (48.0 %) eyes and better than 0.1 in 5 (20.0 %) eyes. There was no statistically significant difference in baseline visual acuity between the two groups (chi-squared test, x^2^ = 2.925, *P* = 0.2316). The visual acuity in the CRVO group after the last follow-up was NLP to CF in 3 (15.8 %) eyes, 0.01–0 .09 in 15 (78.9 %) eyes, and better than 0.1 in 1 (5.3 %) eye. In the PDR group, visual acuity was NLP to CF in 3 (12.0 %) eyes, 0.01–0.09 in 10 (40.0 %) eyes, and better than 0.1 in 12 (48.0 %) eyes. In the PDR group, visual acuity after treatment was better than visual acuity in the CRVO group, and there was a statistically significant difference between the two groups (chi-squared test, x^2^ = 9.669, *P* < 0.05). The proportions of eyes with residual glaucoma after completion of the treatment process were as follows: 6 (31.6 %) eyes continued to receive IOP-lowering medication in the CRVO group, and 6 (24.0 %) eyes continued to receive the same medication in the PDR group. The proportion of eyes with of residual glaucoma in the PDR group was less than the proportion in the CRVO group, and there was no statistically significant difference between the two groups (x^2^ = 0.3126, *P* = 0.5761).

### Typical cases

#### Case 1 (Fig. 3)

**Fig. 3:**
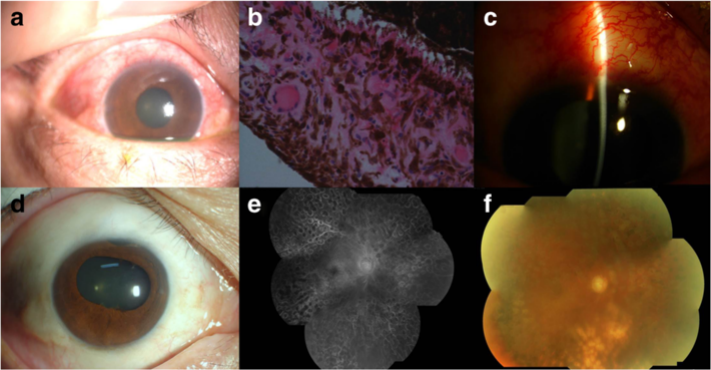
Typical examination results of a 53-year-old man diagnosed with CRVO combined with NVG in the right eye. His physical examination was demonstrated in **a** and **b. c** showed the clinical finding after a trabeculectomy. At the patient’s follow-ups at 12 (**e**) and 24 (**f**) months after discharge, his visual acuity was 0.03 and his IOP was 18 mmHg in the right eye. No leakage was observed in FFA (**d**)

A 53-year-old man presented with blurry vision in the right eye for 6 months and pain for 12 days. The physical examination showed that the visual acuity in his right eye was hand movement. The IOP was 36 mmHg with right corneal edema and iris neovascularization. The pupillary light reflex was slow. Vitreous bleeding and retinal flame-shaped hemorrhage could be observed by fundus examination, but it was not clear. He was diagnosed with CRVO combined with NVG in the right eye and was enrolled into our study for treatment. Medication was administered to control IOP, and this was followed by an intraocular injection of 1.25 mg of Avastin into the right eye. However, the IOP was still beyond control (42 mmHg). Therefore, a trabeculectomy was performed 13 days later, and PRP was carried out starting 10 days after the operation until the completion of PRP at 6 weeks after the surgery. At the patient’s follow-up at 24 months after discharge, his visual acuity was 0.03 and his IOP was 18 mmHg in the right eye. IOP-lowering medications were not applied after this time.

#### Case 2 (Fig. 4)

**Fig. 4:**
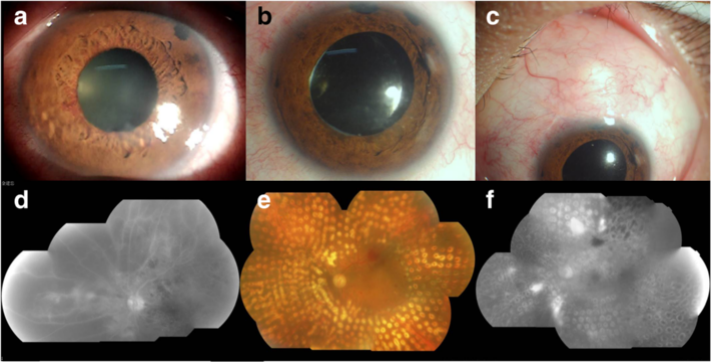
Typical examination results of a 46-year-old woman diagnosed with PDR combined with NVG. **a** Demonstrated in the physical examination. The cornea returned to clear after a trabeculectomy (**b**, **d**). PRP was carried out (**e**). At the end of the follow-up, at 10 months after the patient’s discharge, her visual acuity was 0.05 and her IOP was 20 mmHg in the left eye (**c**). No leakage was observed in FFA leakage (**f**)

A 46-year-old woman with a history of diabetes for 2 months who presented with decreasing visual acuity for 1 month and who had suffered from pain for 14 days was admitted. In the physical examination, the visual acuity in her left eye was 0.03. IOP was 45 mmHg, and corneal edema and iris neovascularization were observed. The pupillary light reflex was slow. Retinal exudation and bleeding spots could barely be observed by fundus examination.

He was diagnosed with PDR combined by NVG in the left eye and enrolled into our study for treatment. First, active medication was administered to control IOP, and this was followed by an intraocular injection of 1.25 mg of Avastin into the left eye. The IOP was still beyond control (38 mmHg), so a trabeculectomy was performed 4 days later, after which the cornea then returned to clear. PRP was performed three times for a total of 3000 points. At the end of the follow-up, at 10 months after the patient’s discharge, her visual acuity was 0.05 and her IOP was 20 mmHg in the left eye. No IOP-lowering medication was applied after this time.

## Discussion

This prospective, interventional study establishes a therapy strategy for NVG as follows. First, the core purpose of all treatments is to lower IOP and preserve the patient’s visual function. Second, anti-VEGF treatment can regress neovascularization at the iris and anterior chamber angle, which allows optimal conditions for intraocular surgery. Third, using anti-glaucoma surgery with or without phacoemulsification or vitrectomy contributes to creating the conditions necessary for the completion of PRP. Last but not least, a change in the current view is needed because the nature of NVG changes after PRP is completed, when NVG changes into general glaucoma. Thus, we can treat residual glaucoma in a general way. In our study, 93.2 % of the patients achieved stable or improved vision, and only three eyes experienced decreased vision. Even more gratifying was that 12 eyes had a visual acuity ≥0.1 after treatment. These results were significant even in patients who had a visual acuity ≤0.1, and especially so for those in whom the other eye was blind. There were five eyes with NLP at the time of diagnosis that lost their light perception (LP) within 1 week. Fortunately, three of these eyes re-acquired some visual acuity after positive treatment. In addition, IOP was well controlled in all of the patients in this study [2, 9, 10, 17].

A deliberate treatment plan must be established according to the conditions of the primary disease and the individual characteristics of NVG. On the one hand, efforts should be made to reduce IOP to protect visual function, while on the other hand, the primary retinal disease should be treated to improve retinal ischemia. Preserving the maximal extent of the patient’s visual function is the core purpose of NVG treatment [7, 11]. This requires doctors to comply with the following three points. First, patient’s IOP must be reduced as soon as possible to minimize the damage caused by a high IOP on visual function. In patients, this can be achieved by applying IOP-lowering drugs as well as anterior chamber puncture, in some cases. If IOP cannot be well controlled by anti-VEGF therapy, anti-glaucoma surgery should be decisively performed to minimize the damage caused by a high IOP. Second, IOP-lowering measures should be performed throughout the treatment process. Close follow-up and monitoring of IOP should be performed until the IOP decreases to a normal level, and it should be kept stable. During this time, medication and surgical treatment options should be regularly adjusted. In our study, ciliary body destructive surgery was not applied out of consideration for the ocular atrophy caused by difficulty with precise quantitation.

Anti-VEGF treatment is an important component of NVG treatment strategies. The advent of anti-VEGF therapies introduced a new concept for treating intraocular neovascular disease by neutralizing VEGF and thereby regressing intraocular neovessels [6, 7, 10, 15]. On the one hand, intraoperative bleeding is not prone to happen during anti-glaucoma surgery, making the success of the surgery possible. On the other hand, anti-VEGF therapy can effectively alleviate disease progression in patients who cannot complete PRP for various reasons. This creates an opportunity to use photocoagulation treatment. Repeated anti-VEGF therapy can be performed if necessary [18–21].

Anti-glaucoma surgery is a significant component in treatments for NVG. Although filtering blebs were, in some patients in this study, no longer obvious a few months after anti-glaucoma surgery, PRP can be confidently completed under conditions including a stable IOP during these months. In other words, anti-glaucoma surgery provides an effective opportunity to complete retinal laser treatment, while avoiding the damage to visual function that can be caused by an increased IOP [8, 10, 13, 22, 23]. The optimal choice is filtration surgery, and glaucoma valve implantation can be performed later if filtration surgery fails; however, the converse of this sequence is impractical because of the formation of conjunctival scarring.

The above measures can not only lower IOP and thereby save visual function, they can also create the conditions necessary for the completion of PRP. The most effective treatment is PRP, the effect of which is to improve the state of retinal blood circulation and prevent it from releasing VEGF. Repressing VEGF contributes to new vessel regression in the iris and angle. Compared to anti-VEGF therapy, PRP radically improves retinal ischemia and thereby inhibits VEGF release, yet anti-VEGF drugs that are intraocularly injected are only retained locally for a short period of time. Therefore, they do not inhibit VEGF release in the long-run [11, 14, 16]. Conditions should be created that allow PRP in cases of refractive media opacity, such as a cataract, corneal edema caused by increased IOP, vitreous hemorrhage, or large-scale preretinal hemorrhage. Active surgery should be performed to remove the opacified media, such as a cataract or vitreous hemorrhage, or complete PRP should be performed during surgery.

## Conclusions

In summary, a comprehensive therapy regimen for NVG that has the preservation of visual function as its core purpose, complete PRP as its goal, and anti-VEGF therapy and anti-glaucoma surgery as its key means can effectively control IOP and retain a portion of a patient’s visual function.

## Abbreviations

anti-VEGF: Anti-vascular endothelial growth factor
CRVO: Central retinal vein occlusion
INV: Iris neovascularization
IOP: Intraocular pressure
IVB: Intravitreal bevacizumab
NVG: Neovascular glaucoma
PDR: Proliferative diabetic retinopathy
PRP: Pan-retinal photocoagulation
